# The Interaction of Pre-programmed Eye Movements With the Vestibulo-Ocular Reflex

**DOI:** 10.3389/fnsys.2018.00004

**Published:** 2018-03-09

**Authors:** Stephanie E. Haggerty, W. Michael King

**Affiliations:** ^1^Department of Biomedical Engineering, University of Michigan, Ann Arbor, MI, United States; ^2^Kresge Hearing Research Institute, Ann Arbor, MI, United States; ^3^Department of Otolaryngology Head and Neck Surgery, University of Michigan, Ann Arbor, MI, United States

**Keywords:** Vestibulo-Ocular Reflex, efference copy, adaptation, biological, internal model, gaze stabilization

## Abstract

The Vestibulo-Ocular Reflex (VOR) works to stabilize gaze during unexpected head movements. However, even subjects who lack a VOR (e.g., vestibulopathic patients) can achieve gaze stability during planned head movements by using pre-programmed eye movements (PPEM). The extent to which PPEM are used by healthy intact subjects and how they interact with the VOR is still unclear. We propose a model of gaze stabilization which makes several claims: (1) the VOR provides ocular stability during unexpected (i.e., passive) head movements; (2) PPEM are used by both healthy and vestibulopathic subjects during planned (i.e., active) head movements; and (3) when a passive perturbation interrupts an active head movement in intact animals (i.e., combined passive and active head movement) the VOR works with PPEM to provide compensation. First, we show how our model can reconcile some seemingly conflicting findings in earlier literature. We then test the above-mentioned predictions against data we collected from both healthy and vestibular-lesioned guinea pigs. We found that (1) vestibular-lesioned animals showed a dramatic decrease in compensatory eye movements during passive head movements, (2) both populations showed improved ocular compensation during active vs. passive head movements, and (3) during combined active and passive head movements, eye movements compensated for both the active and passive component of head velocity. These results support our hypothesis that while the VOR provides compensation during passive head movements, PPEM are used by both intact and lesioned subjects during active movements and further, that PPEM work together with the VOR to achieve gaze stability.

## Introduction

Maintaining a stable line of sight in the midst of head movement is essential to normal function. Indeed, those who suffer from oscillopsia, an inability to stabilize the visual world, can be severely disabled and even incapacitated by their condition (Crawford, 1964; Chambers et al., 1985). During unexpected, or passive, head movements, it is the Vestibulo-Ocular Reflex (VOR) which works to stabilize gaze by producing compensatory eye movements. However, only recently has the question of how gaze is stabilized during planned or active, head movement been raised.

One of the first to study this question was Dichgans et al. (1973) when they investigated what drove gaze stabilization at the end of self-generated eye-head gaze shifts. They systematically eliminated sensory feedback and found that after bilateral vestibular lesions, there was a dramatic decrease in compensatory eye movement. However, they also noted that after a few weeks there was a nearly complete recovery of compensation during planned head movements. Moreover, these compensatory eye movements occurred even when the head movement was unexpectedly blocked, suggesting that they were pre-programmed. The authors hypothesized that during an active head movement, the body can predict how the head will move and thus, pre-program the necessary compensatory eye movements. Yet healthy intact animals did not exhibit this pattern: when head movements were prevented in these subjects, so were the compensatory eye movements. This led to the conclusion that PPEM were an adaptive strategy only developed with the loss of vestibular input, and that in healthy animals, it was the VOR that was primarily responsible for gaze stabilization even during planned head movements. This is in line with what clinicians have observed in vestibulopathic patients. Namely, that while compensatory eye movements during passive perturbations remain insufficient, patients can partially recover gaze stability during active head movements (Foster et al., 1997; Herdman et al., 2001; Tian et al., 2002; Halmagyi et al., 2003; Black et al., 2005).

However, improved compensation during active, as compared to passive, head movements has also been documented in healthy human and non-human subjects both in terms of increased gain (Tomlinson et al., 1980; Collewijn et al., 1983; Van der Steen and Collewijn, 1984; Jell et al., 1988; Hoshowsky et al., 1994) and decreased latency (Della Santina et al., 2002; Shanidze et al., 2010a). As the latency in a healthy subject corresponds to minimum signal transduction time along the VOR pathway (Huterer and Cullen, 2002), a decrease in latency would be evidence for pre-programming. An important difference between these studies and that of Dichgans et al. is how each identified PPEM. Dichgans defined PPEM as compensatory eye movements which occurred even when the planned head movement was prevented. Other authors defined PPEM as eye movements that exhibited improved compensation as measured by gain and latency.

We have developed a model which unifies these seemingly conflicting findings. It suggests that PPEM are indeed a part of normal gaze stabilization resulting in improved compensation during active head movements, similar to what clinicians have observed. It also predicts that if the gain of PPEM and VOR are similar (as is the case for non-human primates and humans), PPEM would not be observed in healthy subjects when planned head movements are prevented, as described by Dichgans et al.

Our model also predicts that while PPEM are the primary means of gaze stability during active head movements, the VOR remains online to compensate for any unexpected passive perturbations. This claim, that the VOR remains operational during voluntary movements, harkens back to an older, but still on-going controversy regarding gaze shifts. Briefly, gaze shifts require the eyes and head move in the same direction; thus, the VOR would seem counterproductive. This has led many to argue that the during gaze shifts, the VOR is suppressed (Laurutis and Robinson, 1986; Tabak et al., 1996; Cullen et al., 2004). Others, however, have found that the VOR remains online and continues to compensate for passive perturbations (Morasso et al., 1973; Blakemore and Donaghy, 1980; Guitton et al., 1984; Guitton and Volle, 1987; Freedman et al., 1998; Bechara and Gandhi, 2010).

Similarly, our hypothesis that PPEM stabilize gaze during active head movements would seem to render the VOR counterproductive as it would interfere with the ongoing PPEM. Thus, VOR suppression (which we will refer to as the “Suppression Model”) presents itself as a plausible mechanism for preventing this interference. However, as will be described in more detail below, our model allows the VOR to compensate for unexpected perturbations while preventing it from interfering with PPEM. We refer to this as the “Cooperative Model” and it is precisely this cooperation between PPEM and the VOR which allows us to reconcile conflicting findings in earlier literature as previously described.

Consequently, our model makes three claims: (1) the VOR provides ocular stability during passive head movements; (2) PPEM are used by both healthy and vestibulopathic subjects during active head movements; (3) during active head movements, the VOR is not suppressed but continues to provide compensation. To test our model, we first compare compensatory eye movements in healthy and lesioned animals during active and passive head movements to confirm the necessity of the VOR during passive head movements and the presence of PPEM during active movements in both populations. We then examine compensatory eye movements that occur when a passive perturbation interrupts an ongoing active head movement. If the VOR is suppressed, we expect these eye movements to reflect only the active component; if the VOR is intact, eye movements should compensate for the total head velocity.

## Model

**Figure 1:**
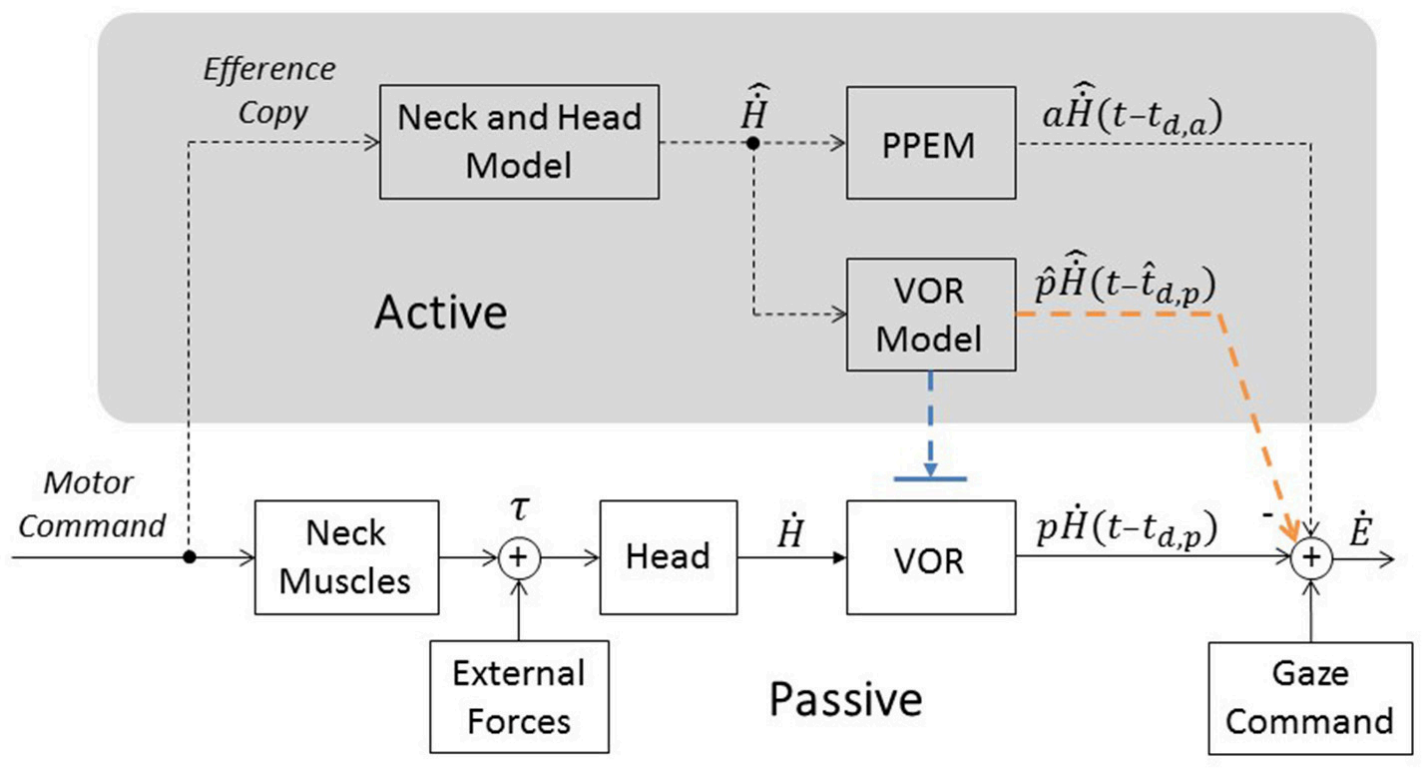
Model of gaze stabilization. Bottom portion represents traditional pathways (i.e., the VOR and Gaze Command). Top portion (in gray, labeled “Active”) includes a pathway that estimates head velocity (“Neck and Head Model”) and necessary pre-programmed eye movements (“PPEM”) and two alternative pathways that interact with the VOR. The Suppression Model, in blue, that turns off the VOR and the Cooperative Model, in orange, that estimates the VOR's response (“VOR Model”) and subtracts it from the total eye movement.

Our hypothesis is that while the VOR pathway provides gaze stabilization during passive head movements, pre-programmed eye movements (PPEM) are used during active head movements. Thus, our model (Figure 1) is composed of two parallel pathways which we denote as “Active” (top) and “Passive” (bottom). We have also included a gaze command input (bottom right) to simulate previous studies which used gaze shifts. During gaze stabilization, however, the gaze command is always zero and can effectively be ignored.

The passive pathway represents the traditional understanding of gaze stabilization. The head is moved by either an external force or a self-generated motor command. In either case, the resulting head velocity (*Ḣ*) is detected and processed by the VOR pathway to produce a compensatory eye movement which can be described by Ė = *pḢ*(*t*−*t*_*d,p*_) where *p* is the passive gain and *t*_*d,p*_ is the passive time delay associated with the VOR pathway, usually 5–7 ms (Huterer and Cullen, 2002; Shanidze et al., 2010a).

We propose that in addition to the passive pathway there is an active pathway that provides enhanced compensation during planned head movements. By taking a copy of the motor command, called an efference copy (von Holst and Mittelstaedt, 1950) and feeding it into an internal model of the head and neck muscles, the body can predict how the head will move ($\hat{Ḣ}$). Based on this prediction, it can pre-program the necessary eye movements which can be described by $Ė=a\hat{Ḣ}\left(t-t_{d,a}\right)$ where *a* is the gain of the PPEM, or the active gain, and *t*_*d,a*_ is the active delay.

As the vestibular afferents detect all head movements, including active ones (Cullen and Minor, 2002), the VOR could produce eye movements that would interfere with PPEM. We propose that to prevent this interference, the active pathway selectively cancels the VOR. To do this, the same prediction of head movement ($\hat{Ḣ}$) used to produce PPEM is also sent to an internal model of the VOR. This model predicts the eye movements the VOR will produce according to the formula $Ė=\hat{p}\hat{Ḣ}\left(t-{\hat{t}}_{d,p}\right)$, where $\hat{p}$ is the estimate of the passive gain and ${\hat{t}}_{d,p}$ is the estimate of passive delay (Figure 1, orange pathway). This prediction is then subtracted from the actual output of the VOR. Thus, the VOR is prevented from interfering with PPEM but can still provide compensation for unexpected passive perturbations. We refer to this model as the Cooperative Model. However, as discussed in the earlier, an alternative mechanism for preventing VOR interference is simply to suppress the VOR altogether during active movements (Figure 1, blue pathway) which we refer to as the Suppression Model.

In this paper, we will evaluate eye movements during three types of head movements: (1) passive-only, (2) active-only, and (3) combined active and passive, where a passive perturbation interrupts an active movement. Below are the predictions made by each model.

(1) During a passive-only movement, when $\hat{Ḣ}=0$, both models predict

(1) $$\dot{E}=p*\dot{H}(t-t_{d,p})$$

(2) During an active-only movement, when $Ḣ=\;\hat{Ḣ}$, the Cooperative model predicts

(2) $$\dot{E}=p*\dot{H}\left(t-t_{d,p}\right)-\hat{p}*\dot{H}\left(t-{\hat{t}}_{d,p}\right)+a*\dot{H}(t-t_{d,a})$$

For animals that can correctly estimate their VOR dynamics (i.e., $\hat{p}=p,\;{\hat{t}}_{d,p}=t_{d,p}$), this reduces to the following formula:

(3) $$\dot{E}=a*\dot{H}(t-t_{d,a})$$

According to the Cooperative Model, we use Equation (3) for healthy intact animals. However, for animals that have recently undergone a change in VOR dynamics (such as a vestibular lesion) Equation (2) is more appropriate as it can take time for the internal model to update its estimate of the VOR dynamics and thus $\hat{p}\neq p$ and ${\hat{t}}_{d,p}\neq t_{d,p}$.

(3) During a combined head movement, the Cooperative Model predicts

(4) $$\dot{E}=p*\dot{H}\left(t-t_{d,p}\right)-\hat{p}*\hat{\dot{H}}\left(t-{\hat{t}}_{d,p}\right)+a*\hat{\dot{H}}(t-t_{d,a})$$

However, in our analysis, we often refer to the “active” and “passive” component of head movement rather than the actual (*Ḣ*) and predicted ($\hat{Ḣ}$) head movement. To make these ideas clearer, we reformulate Equation (4) in those terms.

We can think of the predicted head movement ($\hat{Ḣ}$) as the active component of head movement, as it is the head movement expected to result from the active motor command. We will thus denote $\hat{Ḣ}$ as Ḣ_*a*_. The total head velocity (Ḣ) can be thought of as the sum of the active and passive component (Ḣ_*a*_ + Ḣ_*p*_). Making these substitutions and rearranging, we find that in a healthy intact animal ($\hat{p}=p,\;{\hat{t}}_{d,p}=\;t_{d,p}$), Equation (4) becomes

(5) $$\dot{E}=p*{\dot{H}}_{p}(t-t_{d,p})\;+a*{\dot{H}}_{a}(t-t_{d,a})$$

Alternatively, the Suppression Model predicts:

(6) $$\dot{E}=a*{\dot{H}}_{a}(t-t_{d,a})$$

Note, the distinguishing feature of Suppression Model is that eye movements reflect only the active component of head movement.

To simulate results from Dichgans and others (Dichgans et al., 1973; Newlands et al., 1999; Sadeghi et al., 2012) we used the following parameters. Intended head velocity ($\hat{Ḣ}$) was always set to a decaying exponential waveform, designed to emulate the head movement reported by Dichgans et al. Actual head velocity was set equal to the intended head velocity (i.e., $Ḣ=\;\hat{Ḣ}$) when the head was free to move and set to zero when head movement was prevented. For PPEM, we used an active gain (*a*) of −1.0 and a latency (*t*_*d, a*_) of 0 ms to simulate ideal compensation. For the VOR, we used a passive gain (*p*) of −1.0 for healthy animals (Dichgans et al., 1973; Newlands et al., 1999, 2001; Huterer and Cullen, 2002) and 0.0 for lesioned animals (Dichgans et al., 1973; Newlands et al., 1999; Sadeghi et al., 2012) and latency (*t*_*d,p*_) of 5 ms (Huterer and Cullen, 2002). For the VOR Model, we assume that in a healthy adult animal, the VOR dynamics are stable and can be accurately estimated. We, therefore, set the estimated passive gain and latency ($\hat{p},\;{\hat{t}}_{d,p}$) equal to the actual passive gain and latency. After a labyrinthectomy, we assume that the VOR Model initially maintains its original dynamics, but with time, these change to reflect the animal's new state. What this new state is depends on a number of factors including the severity of the lesion and compensation from other systems. In Dichgans' study, the authors found that while the passive VOR gain was effectively zero after lesion, the cervical-ocular reflex (COR) provided about 30% ocular compensation. Given the lack of the COR before lesioning, we assume that the COR is interpreted as the passive VOR and therefore set estimated passive VOR gain to −0.3. For animals that also underwent cervical deafferentation, and therefore had no VOR or COR, we set the estimated passive gain to 0.0.

## Experimental methods

### Surgical preparation

All procedures were approved by the University of Michigan's University Committee on Use and Care of Laboratory Animals and were in accordance with the National Institutes of Health Guide for the Care and Use of Laboratory Animals. Seven male pigmented guinea pigs were used for this study. All animals underwent an initial surgery during which a head bolt and eye coil were implanted. Briefly, animals were sedated using a combination of ketamine and xylazine. A midline incision was made to expose the skull and a head post was attached using bone cement and dental acrylic. Next, an eye coil was implanted under the conjunctiva of the right eye. Animals were allowed to recover for 7–14 days before data was collected.

In addition, 3 animals underwent bilateral vestibular lesions after control data was collected. Lesions were performed by filling the inner ear cavity with streptomycin. Lesions were performed one at a time. That is, each animal first underwent a unilateral lesion and, after approximately 1 month, underwent the same procedure on the contralateral side.

### Test procedure

Animals were comfortably restrained to a turn table, such that their bodies were fixed to the turn table but their heads were free to move (for details, Shanidze et al., 2010b). Eye movements were recorded via a scleral eye coil (see Surgical Preparation for details). Head movements were recorded via two orthogonal coils imbedded in a light-weight plastic ball which attached to the head post. All procedures were performed in the dark.

Passive stimuli consisted of transient velocity steps of 60 deg/s generated by the turn table. Using a Gaussian acceleration profile, maximum step velocity was reached after 90 ms, lasted for approximately 400 ms, and then decelerated in a similar manner. Each testing session consisted of at least 100 steps and active movements were encouraged throughout testing by placing food in eccentric locations. Control animals were tested between 5–9 times while lesioned animals were tested 14–20 times.

### Data analysis

Orientation data from eye and head coils was differentiated and low-pass filtered at 40 Hz to obtain gaze (*Ġ*) and head (*Ḣ*) velocity in space. Eye (Ė) velocity in the head was defined as *Ė* = *Ġ* − *Ḣ*. The velocity feedback signal from the turntable was defined as body velocity (*Ḃ*) and head-on-body ($\overset{{}^{\circ}}{HoB}$) velocity was defined as $\dot{HoB}=Ḣ-Ḃ$.

**Figure 2:**
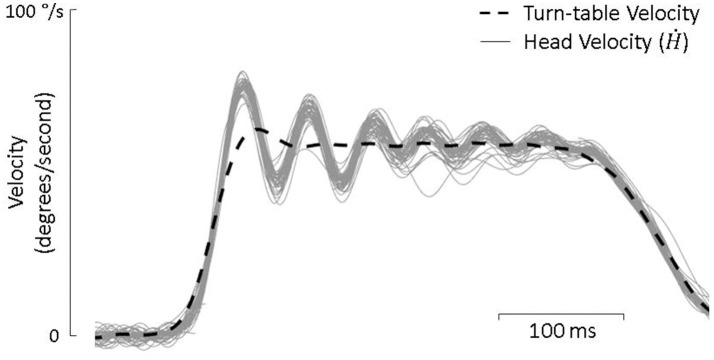
Turn-table velocity and head velocity from each of the approximately 80 passive-only trials during a single test day. The head consistently responds as a underdamped second order system with decaying oscillations.

Active-only head movement was defined as head movement that surpassed 10 deg/s continually for 200 ms in the absence of any turntable movement. In total, there were 225 min of active movement from control animals and 220 min from lesioned animals that were included in the analysis. Passive-only movements were defined as head movements that occurred in response to turn table velocity steps in the absence of any additional active head movement. When a velocity step interrupted an on-going active movement, the head movement was defined as a combination of active and passive. In these cases, we defined the passive component (*Ḣ*_*p*_) of head movement not as the turn-table velocity but as the averaged passive-only response for that day (Brooks and Cullen, 2014). This is because the head does not follow the turn table velocity but rather responds as a second-order system with exponential decaying oscillations (see Figure 2) which are nonetheless consistent and can be predicted by the passive dynamics of the head and neck (Peng et al., 1996, 1999). Figure 2, for example, shows roughly 80 repetitions of passive-only head perturbations with little variation between trials. Meanwhile, the active component of head movement (*Ḣ*_*a*_) was defined as the total head velocity (*Ḣ*) minus this averaged response. For both control and lesioned animals, there were approximately 3,500 passive-only steps and 600 combined active and passive steps.

As other dynamics and reflex pathways can contribute to eye movement, we restricted our analysis to the first 65 ms of head movement in all conditions defined above. All fits were performed using least-squares regression. To determine the gain and latency for active- and passive-only movements, we used the following fit (Huterer and Cullen, 2002):

(7) $$\dot{E}=offset+g*\dot{H}(t-t_{d})$$

Similar to Equations (1) and (3), where *g* is either the active (*a*) or passive (*p*) gain, *Ḣ* is the total head velocity, and *t*_*d*_ is either the active (*t*_*d,a*_) or passive (*t*_*d,p*_) latency. The only exception to this was for lesioned animals during active-only movements where the assumptions that $\hat{p}=p$ and ${\hat{t}}_{d,p}=t_{d,p}$ do not hold. Thus, these movements were fit according to Equation (2):

(8) $$\dot{E}=offset+p*\dot{H}(t-t_{d,p})-\hat{p}*\dot{H}\left(t-{\hat{t}}_{d,p}\right)+a^{*}\dot{H}(t-t_{d,a})$$

For each animal and each day, fits were first performed to passive-only and active-only data using Equations (7) and (8) accordingly. Parameters estimated with those fits (i.e., $\;p,t_{d,p},\;a,t_{d,a},\;\hat{p},{\hat{t}}_{d,p}$) were used for fits to combined active and passive movements. For these movements, the Cooperative Model predicted eye movements described by Equation (5) for healthy animals and Equation (4) for lesioned animals, thus we used Equations (9) and (10) respectively:

(9) $$\dot{E}\;=offset+p*{\dot{H}}_{p}(t-t_{d,p})+a*{\dot{H}}_{a}(t-t_{d,a})$$

(10) $$\begin{array}{l} \dot{E}=offset+p*{\dot{H}}_{p}(t-t_{d,p})+p*{\dot{H}}_{a}(t-t_{d,p})-\hat{p}*{\dot{H}}_{a}\left(t-{\hat{t}}_{d,p}\right)\\ \;+a*{\dot{H}}_{a}(t-t_{d,a}) \end{array}$$

While the Suppression Model predictions were described by Equation (6) for both populations:

(11) $$\dot{E}=offset+a*{\dot{H}}_{a}(t-t_{d,a})$$

To compare these two models, we first used the coefficient of determination (*R*^2^). However, as the two models have different number of parameters and *R*^2^ is known to increase with added parameters, we also used the Bayesian Information Criterion (BIC) which is considered one of the most conservative model selection criterion (Schwarz, 1978; Posada et al., 2004) and is defined as:

(12) $$BIC=n*\ln\left(\frac{SSE}{n}\right)+k*\ln(n)$$

Where *n* is the number of observations, *SSE* is the sum-squared errors, and *k* is the number of parameters. A lower BIC indicates a better fit.

Statistical significance was assessed using a paired *t*-test with alpha = 0.05.

## Results

In Dichgans original paper, PPEM were defined as compensatory eye movements seen when a planned head movement was unexpectedly prevented. They found that while PPEM were observed in animals within a few days of vestibular lesion, they were not observed in healthy subjects, leading them to conclude that PPEM were strictly an adaptive phenomenon. We suggest that our model can account for these findings despite our inclusion of PPEM as a part of normal gaze stabilization.

**Figure 3:**
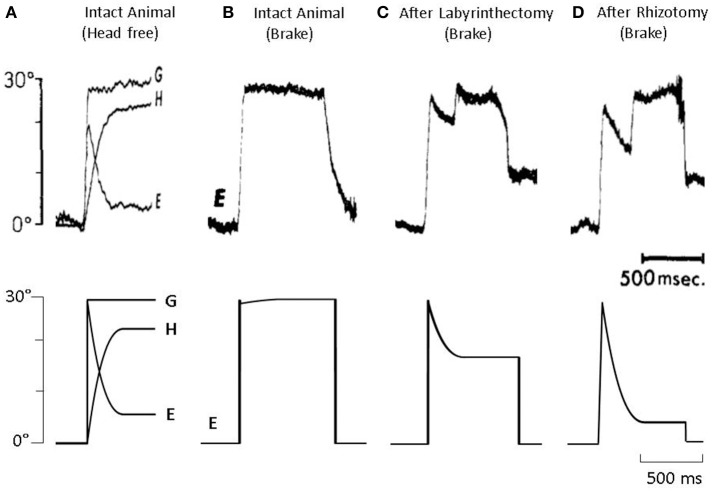
(Top) Data from Dichgans et al. (1973) and (bottom) model simulations. **(A)** Gaze (G), head (H), and eye (E) position traces from an intact animal during a voluntary head rotation. **(B)** Eye position from an intact animal when a voluntary head rotation is unexpectedly stopped via head brake. **(C)** Eye position from a vestibular-lesion animal, that also underwent cervical deafferentation, when a voluntary head rotation is unexpectedly stopped. **(D)** Eye position from a vestibular-lesion animal, that also underwent cervical deafferentation, when a voluntary head rotation is unexpectedly stopped.

In Figure 3, we present four results from Dichgans et al. (1973) paper (top) along with simulations from our model (bottom). Starting on the far left, in panel A, gaze (G), head (H), and eye (E) position traces are presented for a healthy animal during a planned head rotation. As can be seen, the eye (E) makes an immediate saccade to about 20 degrees, then counter-rotates equal and opposite to the head (H), stabilizing gaze (G) in both experimental data and model simulations. In panel B, the planned head rotation is unexpectedly prevented using a head brake. Thus, there is no counter-rotation of the eye as there is no rotation of the head. However, when this same paradigm is used with a vestibular lesioned animal, as seen in panel C, the eye begins to counter-rotate despite there being no head rotation. In animal data, this counter-rotation lasts for approximately 300 ms, at which point the animal makes a refixation saccade. Our model does not include a saccade mechanism. As such, the model correctly predicts the eye counter-rotation, but the eye remains at its new position and does not refixate. Finally, in an animal that has undergone both a labyrinthectomy and cervical deafferentation (panel D), there is an even greater counter-rotation of the eye in both experimental data and model simulations. Thus, in all four examples, the model was able to predict the experimental data using PPEM as part of normal gaze stabilization.

**Figure 4:**
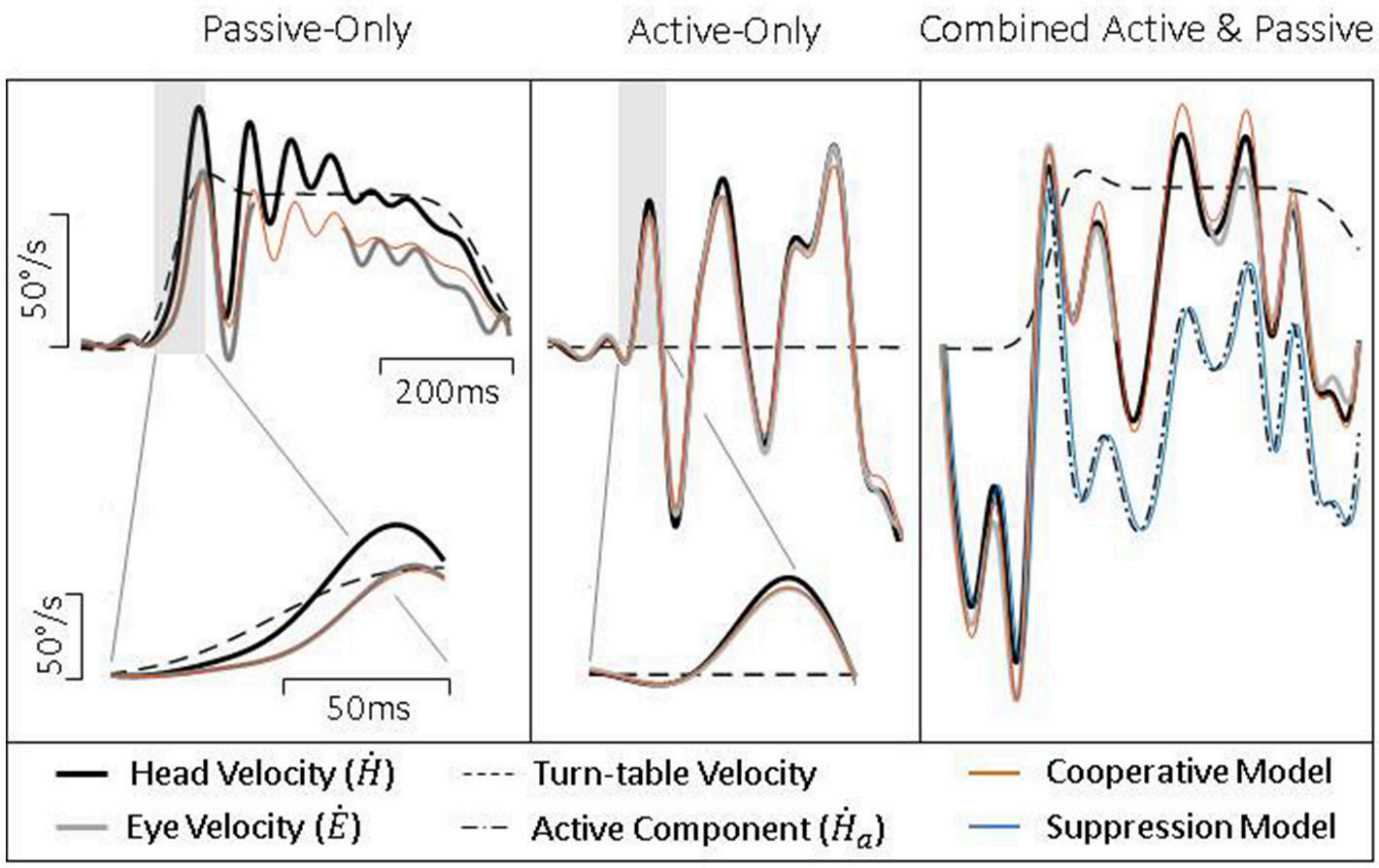
Exemplary data from healthy guinea pigs during passive **(Left)**, active **(Middle)**, and combined **(Right)** head movements. Details show difference in gain and latency of eye movements during passive and active head movements. Model predictions are identical for passive- and active-only movements, but can be distinguished during combined movements.

To further test our model, we collected head and eye movement data from both healthy and vestibular lesioned guinea pigs in order to characterize compensatory eye movements during passive, active, and combined active and passive head movements. Figure 4 presents exemplary data from healthy intact animals. During a passive head rotation (left panel) the guinea pig produces compensatory eye movements that are delayed and diminished with respect to the head (see inset for detail). However, during active head movements (middle panel), eye movements show an increase in gain and decrease in latency, in line with the hypothesis that these are PPEM. When a passive perturbation interrupts an active movement (i.e., combined movement, right panel) the eye appears to compensate for the total head velocity as is expected by our model. To further demonstrate this point, we present in this figure predictions from our model (“Cooperative Model” in orange) which allows the VOR to continue to compensate during active head movements; as well as the alternative hypothesis (“Suppression Model” in blue) in which the VOR is suppressed during active head movements. During passive-only and active-only head movements these two models make the same predictions. However, during combined active and passive head movements these two models clearly differentiate themselves with the Cooperative Model more closely aligned with the actual eye movement.

**Figure 5:**
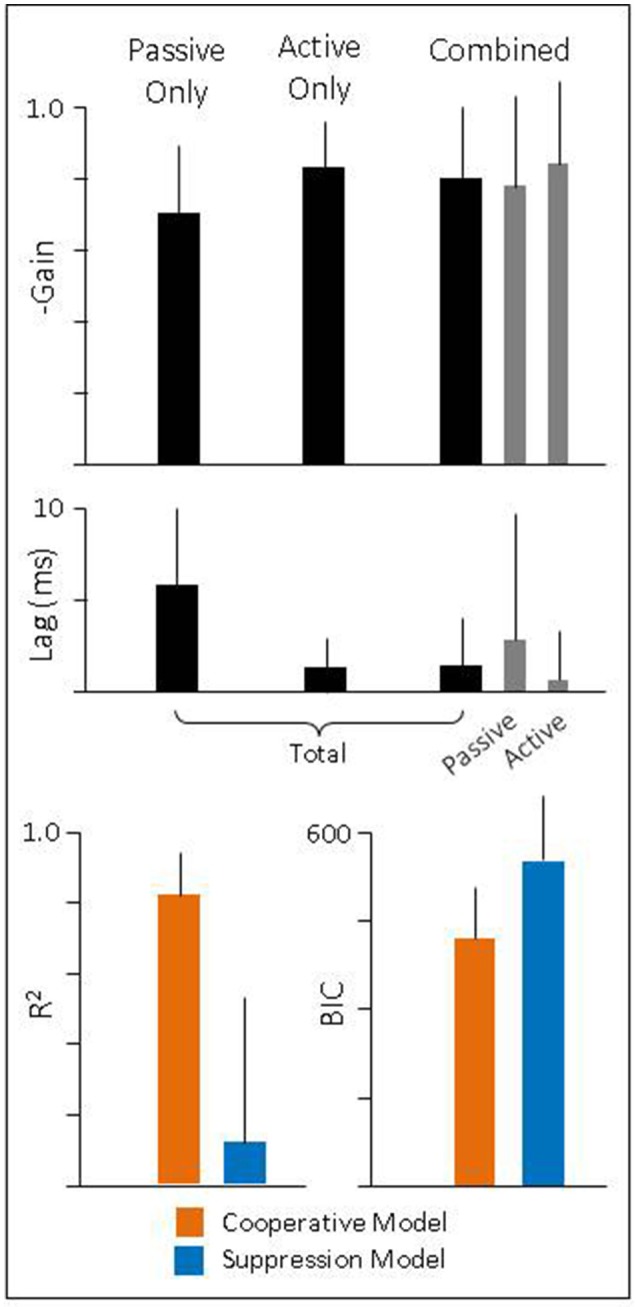
**(Top** and **Middle)** Gain and latency of eye movements during passive, active, and combined head movements. Regressions were performed against total head velocity (black bars, Equation 6) and against passive and active components of head velocity independently (gray bars, Equation 7). **(Bottom)** Goodness of fit for each model.

To quantify these findings, we regressed eye velocity against the total head velocity (Equation 7) for passive-only, active-only, and combined head movements (Figure 5, black bars). For combined head movements, we also regressed eye velocity against the passive and active component of head velocity independently (Equation 8) to determine how each component was being used (Figure 5, gray bars). During passive-only movements we found the VOR had a gain of −0.70 (± 0.19) and latency of 5.8 ms (± 4.2). Both values are in line with what has been previously reported for this species (Escudero et al., 1993; Serafin et al., 1999; Shanidze et al., 2010b). We also found that despite a relatively low gain, eye movements show a strong correlation to head movement, as demonstrated by a high *R*^2^ (0.98 ± 0.01). During active-only movements, compensatory eye movements had a similarly high *R*^2^ (0.97 ± 0.03) but a significantly higher gain (−0.83 ± 0.13; *p* < 0.001) and shorter latency (1.3 ms ± 1.6; *p* < 0.001) as would be expected with PPEM and as previously noted by others (Della Santina et al., 2002; Shanidze et al., 2010a). Thus, despite the goal of stabilizing gaze, gaze moved at approximately 30% of head velocity during passive-only movements but only 17% of head velocity during active-only head movements.

During combined active and passive movements, we found that when eye movements were regressed against total head velocity they had a gain of −0.80 (± 0.20) and latency of 1.4 ms (± 2.6). We also regressed eye movements against the active and passive component to quantify the gain and latency associated with each. We found the passive gain was −0.78 (± 0.26) while the active gain was −0.84 (± 0.23). In addition, the latency associated with the passive component was 2.9 ms (± 7.0) and 0.7 ms (± 2.6) for the active component. These, results mirror those seen during passive- and active-only head movements, namely, that the active component has a higher gain and shorter latency than that of the passive component.

These results would suggest that during active head movements the VOR does continue to compensate for passive perturbations. To explicitly test this theory, we compared our model (“Cooperative”) against the alternative (“Suppressive”) which proposes that the VOR is suppressed during planned movements. We first examined the goodness of fit of each model (Figure 5, bottom panel) and found that the *R*^2^ was higher for the Cooperative model (0.81 ± 0.13) compared to the Suppressive model (0.12 ± 0.41). Next, we calculated the Bayesian Information Criteria (BIC), which takes into account the number of parameters in a model (see Methods for more detail). According to this metric a lower score indicates a better fit to the data. We found that the BIC for the Suppression model was (555 ± 101) greater than that of the Cooperative model (420 ± 87) indicating that the Cooperative model provides a much better description of the data.

We also performed this analysis on lesioned animals to test two further predictions of our model. First, that despite no longer having a functional VOR, PPEM would still be present during active head movements. Second, that given the lack of a VOR in these subjects, the two models would do equally well in predicting eye movements.

**Figure 6:**
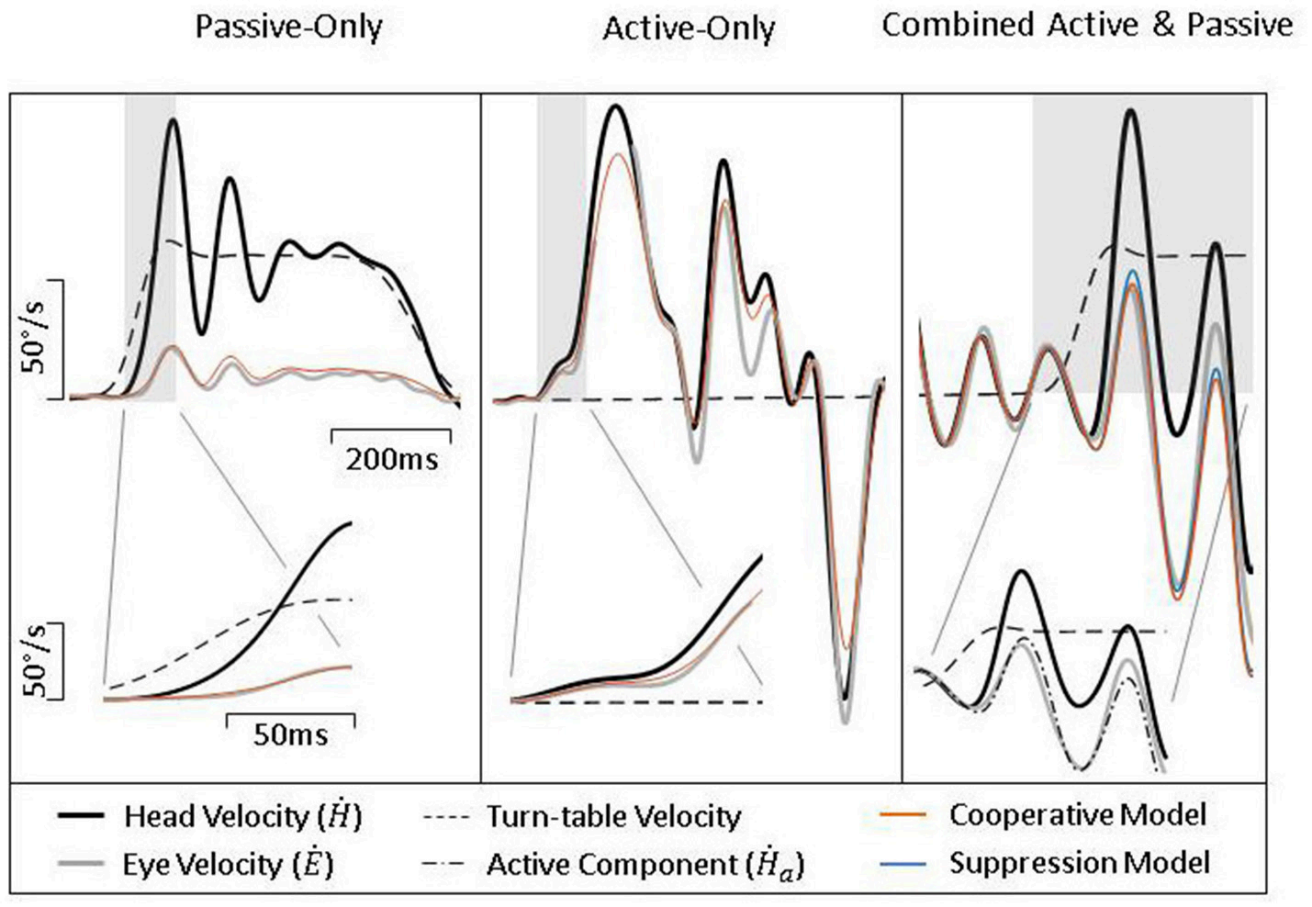
Exemplary data from lesioned guinea pigs during passive **(Left)**, active **(Middle)**, and combined **(Right)** head movements. Details show difference in gain and latency of eye movements during passive and active head movements. Model predictions are identical for passive- and active-only movements, but can be distinguished during combined movements.

Figure 6 shows exemplary data from lesioned animal during passive, active, and combined head movements. As would be expected from a lesioned animal, there is limited ocular compensation during a passive perturbation (left panel). However, during active head movements (middle panel), there is a robust counter-rotation of the eye, similar to that seen in intact animals (Figure 4, middle panel). When a passive perturbation interrupts an ongoing active movement (right panel), we see a very different pattern of eye movement than that seen in healthy animals. In lesioned animals, the eye appears to primarily follow the active component, as can be seen in the inset and fails to compensate for the passive perturbation. This can also be seen in how closely the two models align. The Suppression Model, despite only taking into account the active component of head velocity, makes a similarly accurate prediction of eye velocity as does the Cooperative Model, which takes into account both the active and passive component, indicating that the eye is only compensating for the active component.

To quantify these findings, we performed a similar analysis as described above for intact animals. We found that during passive-only head movements, lesioned animals have a VOR gain of −0.24 (± 0.19) and latency 7.4 ms (± 8.6). Thus, there was still some amount of ocular compensation (as can be seen in Figure 6, left panel); however, it was greatly reduced from the pre-lesion VOR gain of −0.7. We also found that these eye movements have a high correlation to head movement 0.77 (± 0.3) despite their low gain. During active-only head movement, lesioned animals produce compensatory eye movements with a gain of −0.55 (± 0.24) and latency 3.7 ms (± 2.5) with a similarly high correlation to head movement (0.93 ± 0.06). This is consistent with the pattern seen in intact animals, namely, that compensatory eye movements during active head movements have a higher gain and shorter latency, in line with the theory that these eye movements are pre-programmed. For combined active and passive head movements, we found that the passive gain was −0.44 (± 0.24) while active gain was −0.61 (± 0.28) and passive lag was 3.1 ms ± 8.1 while active lag was 1.7 ms ± 4.2. Again, mirroring the pattern seen in intact animals of having a higher gain and shorter latency associated with the active, compared to the passive, component.

To determine which model of VOR and PPEM integration performed better, we again focus on combined active and passive head movements. We found that as in intact animals, the Cooperative model was a better fit to the data than the Suppression model as demonstrated by a higher *R*^2^ (0.50 ± 0.3 vs. 0.36 ± 0.3) and a lower BIC (435 ± 85 vs. 490 ± 117). However, as will be discussed in more detail below, the differences in performance between these two models is greatly diminished in lesioned animals.

## Discussion

The role of PPEM as an adaptive mechanism has been explored in a number of species (human: Kasai and Zee, 1978; Foster et al., 1997; Herdman et al., 2001; Della Santina et al., 2002; Tian et al., 2002; Halmagyi et al., 2003; Black et al., 2005; non-human primate: Newlands et al., 1999, 2001; rodent: Shanidze et al., 2010a; Sadeghi et al., 2012) since it was first reported in primates by Dichgans et al. (1973). These, studies note the occurrence of compensatory eye movements in vestibular deficient subjects during active, but not passive, head movements. Recently, some studies have also noted improved compensation (i.e., increased gain and decreased latency) of eye movements during active head movements in healthy animals as well, suggesting the use of PPEM (human: Tomlinson et al., 1980; Collewijn et al., 1983; Jell et al., 1988; Hoshowsky et al., 1994; Della Santina et al., 2002; rodent: Van der Steen and Collewijn, 1984; Shanidze et al., 2010a). If PPEM are a part of normal gaze stabilization, two questions naturally arise, which this paper seeks to address: first, why didn't Dichgans et al. make the same observation, and second, what happens to the VOR during active movements.

If the VOR remains fully functional it would inevitably produce its own compensatory eye movements, which would interfere with the PPEM. We hypothesized that the efference copy signal used to drive PPEM could also be used to predict the VOR. This prediction could then be used to negate the eye movements the VOR would produce in response the planned head movement (Figure 1, “Cooperation Model”). This would prevent the VOR from interfering with PPEM but allow it to compensate for unexpected head movements. However, it is also possible, and perhaps a simpler explanation, that during active head movements, the VOR is completely suppressed (“Suppression Model”).

To test these two possibilities, we collected data from seven healthy animals and three lesioned animals. Both models use the VOR for passive-only movements and PPEM for active-only head movement, they differentiate themselves when a passive perturbation interrupts an active movement (what we call a “combined movement”). In the Suppression Model, the VOR is completely suppressed and thus, cannot offer compensation. Whereas the Cooperation Model only cancels the VOR with respect to the intended head movement and so can offer compensation for unexpected perturbations. As such, we hypothesized that the Cooperation Model would offer a superior fit to the data in healthy animals but in lesioned animals, which lack a VOR, the two models would make similar predictions.

As can be seen in Figure 4 (left and middle panel), compensatory eye movements in healthy animals have a higher gain and shorter latency during active comparted to passive head movements, confirming earlier reports of the use of PPEM in healthy animals. These results are summarized in Figure 5. When a passive perturbation interrupted an active movement, we found that the eye followed the total head velocity rather than just the active component (Figure 4, right panel) as predicted by the Cooperative Model. To quantify this observation, we compared the goodness of fit (*R*^2^) as well as the BIC for each model. We found that the Cooperative Model had both a higher *R*^2^ (0.81 vs. 0.12) as well as a lower BIC (420 vs. 555) indicating a superior fit to the data.

**Figure 7:**
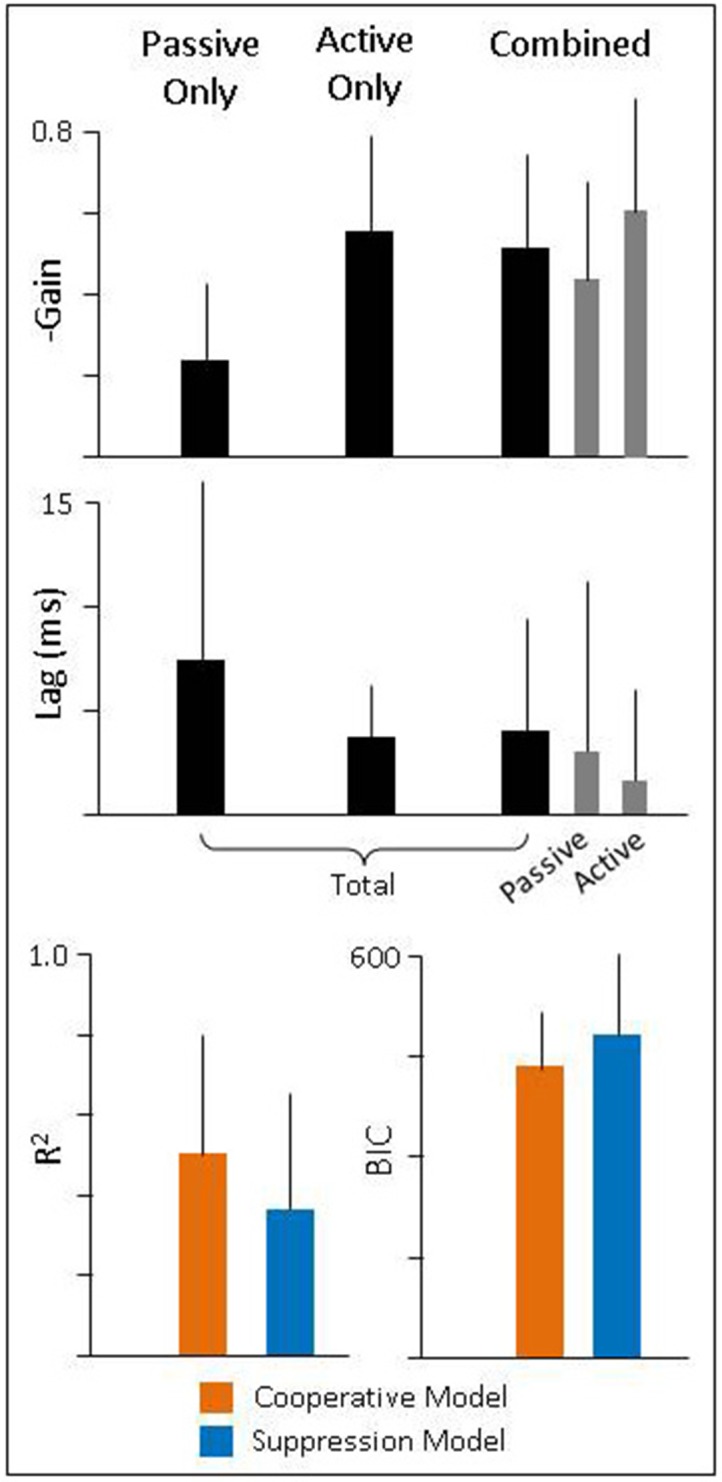
**(Top** and **Middle)** Gain and latency of eye movements during passive, active, and combined head movements. Regressions were performed against total head velocity (black bars, Equation 6) and against passive and active components of head velocity independently (gray bars, Equation 7). **(Bottom)** Goodness of fit for each model.

**Figure 8:**
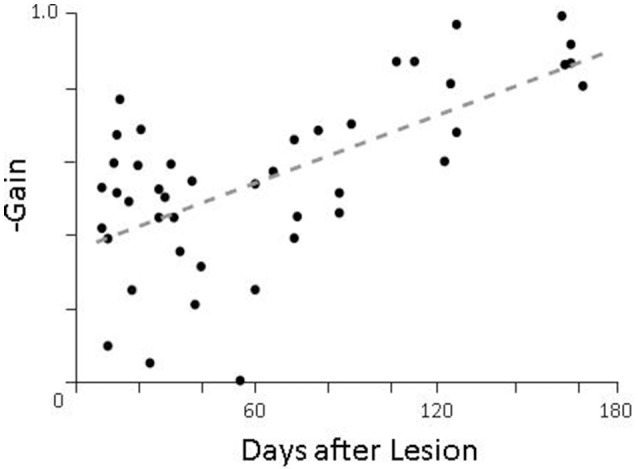
Gain of pre-programmed eye movements as a function of time after lesion. On days when more than one animal was tested, the gains of all animals were averaged together.

For lesioned animals, we found very little compensation during passive movements (Figure 6, left panel) as would be expected, but robust compensation during active head movements (Figure 6, middle panel; summarized in Figure 7). While PPEM were intact after lesion, there was a noticeable decrease in gain (−0.83 vs. −0.55). To investigate this finding, we examined the gain of PPEM over the course of several months (Figure 8) and found that, as Dichgans found in primates, the gain gradually increases. Thus, while the gain of PPEM eventually returns to its original value, taking an average of all data over the post-lesion recovery would result in an overall lower mean gain.

Finally, we found that during combined head movements, eye movements in lesioned animals predominately follow the active component of head velocity and thus, both models offer similar fits (Figure 6, right panel). When we compare the goodness of fit of these two models, we found that while the Cooperative Model still has a higher *R*^2^ (0.50 vs. 0.36) and lower BIC (435 vs. 490), the difference between the two models was much lower. We suspect the Cooperation Model is still a superior fit in our lesioned animals because there was some residual vestibular function, as evidenced by a non-zero VOR gain (Figure 7). As can be seen in Equations (5) and (6), the difference between the two models is a function of the passive gain and they are only equivalent when the gain is zero. Nonetheless, it is interesting to note that the passive gain in lesioned animals was decreased by a little more than half of that in healthy animals (−0.7 vs. −0.24), and the difference in performance between the two models was also decreased by about half (*R*^2^: 85 vs. 30% difference; BIC: 32 vs. 13% difference), in agreement with model predictions.

We were also interested in resolving conflicting reports in the literature as to whether or not PPEM were a part of normal gaze stabilization. Those who reported that PPEM were only found in lesioned animals did so because when the head was unexpectedly stopped, lesioned animals produced compensatory eye movements as though the head had moved, whereas healthy animals did not. However, we found that our Cooperative Model was able to produce both the PPEM observed in lesioned animals as well as the “lack” of such eye movements in healthy animals despite including PPEM for both populations (Figure 3). Two key features of our model that allow for this reconciliation are: (1) that the VOR is selectively cancelled such that it will only provide compensation to unexpected head movements, and (2) what the body expects from the VOR is based on an estimated passive gain ($\hat{p}$) which can be updated. According to this understanding, the eye movement produced during a voluntary head movement is defined as:

(13) $$\dot{E}=p*\dot{H}+\left(a-\hat{p}\right)*\hat{\dot{H}}$$

Using Dichgans methodology, the actual head velocity would be zero (*Ḣ* = 0) and the active gain is ideal (*a* = −1) thus the gain of the PPEM reduces to:

(14) $$PPEM=\;\left(-1-\hat{p}\right).$$

**Figure 9:**
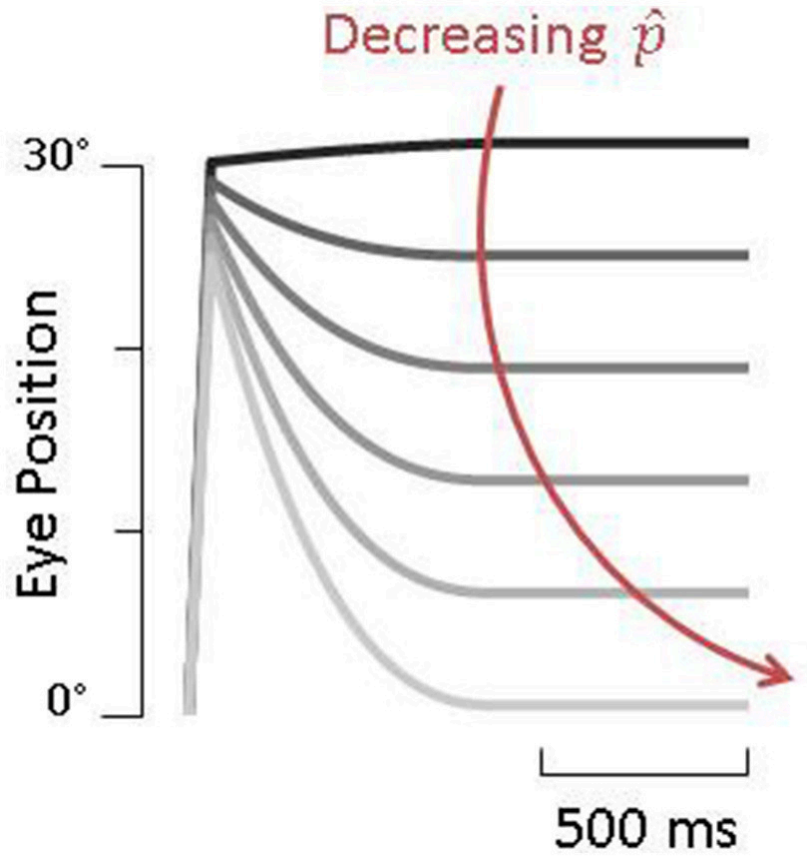
Simulations of pre-programmed eye movements as a function of $\hat{p}$. Darker traces are simulations with higher values of $\hat{p}$ and result in very little compensation whereas lighter traces, with lower values of $\hat{p}$, show large pre-programmed eye movements.

We assume a healthy animal can accurately estimates its passive gain ($\hat{p}=-1$), thus the gain of the PPEM would be zero, that is, no eye movement would be produced, just as described by Dichgans (Figure 3B). In a vestibular lesioned animal, we assume the estimated passive gain would initially be the pre-lesion value ($\hat{p}=-1$) but with time would decrease to match the new passive gain, resulting in a gradual increase in PPEM gain (Figure 9). This fits well with Dichgans finding that the amplitude of PPEM increased with time. They also report that after cervical deafferentation, the PPEM gain further increased. There are two potential explanations for this: (1) cervical deafferentation always occurred after labyrinthectomy, thus the increase in PPEM gain was simply the continued decrease in estimated passive gain; or (2) the COR gain, which in healthy animals is effectively zero but in lesioned animals was −0.3, could have been interpreted as the passive VOR gain, causing the estimated passive gain to settle at a non-zero value. After cervical deafferentation, the COR disappears and thus the estimated passive gain decreases to zero. We based our simulations on this latter interpretation and found that model predictions matched well with the experimental data reported by Dichgans (Figures 3C,D).

As mentioned in the Introduction, the idea of the VOR being selectively cancelled finds its origins in Bizzi's linear summation theory which he proposed to account for the lack of VOR counter-rotation during eye-head gaze shifts. This theory has found support in Bizzi's own studies (Morasso et al., 1973) as well as those of others (Blakemore and Donaghy, 1980; Guitton et al., 1984; Guitton and Volle, 1987; Freedman et al., 1998). In addition, there is research to support a neural mechanism for this phenomenon. Recordings performed by Cullen and colleagues have shown that VO neurons will encode head velocity only during passive head movements (Roy and Cullen, 1998, 2001), despite receiving the entire head velocity signal from primary afferents (Cullen and Minor, 2002). Furthermore, when an active movement occurs in the midst of passive movement, VO neurons encode only the passive component (Cullen et al., 2011) suggesting a selective cancellation. They have proposed a model that is structurally similar to ours, namely, that during active movement, the body can predict the resulting sensory feedback and subtract this from the total head velocity signal from the periphery, leaving only the passive component. They have even reported a signal coming from the cerebellum which corresponds to this prediction (Brooks and Cullen, 2013).

However, there is also evidence, both in eye movements and in single unit recordings, that the VOR is suppressed during active movements. In response to Bizzi's original paper, several studies have shown that when a subject's head is perturbed during the gaze shift, there is no compensation, indicating that the VOR has been turned off (Laurutis and Robinson, 1986; Tabak et al., 1996; Cullen et al., 2004). Yet, this methodology has produced mixed results with some reporting only partial suppression or none at all (Morasso et al., 1973; Blakemore and Donaghy, 1980; Guitton et al., 1984; Guitton and Volle, 1987; Freedman et al., 1998). In recording from larval *Xenopus* frogs, Straka and colleges have shown that compensatory eye movements during locomotion originate from a central pattern generator (CPG) in the spinal cord (Lambert et al., 2012) and not from the vestibular periphery. Further, they have found that the gain of the incoming vestibular signal is significantly decreased during locomotion (Chagnaud et al., 2015). To what extent CPGs are used in higher-level animals, if at all, remains controversial. While there is evidence that they are used for more rhythmic movements like locomotion (Brandt et al., 1999; Jahn et al., 2000), it is unlikely that they are used for the spontaneous unstructured head movements we have described here.

A similar controversy arises when the head is perturbed via microstimulation. Quessy and Freedman (Freedman and Quessy, 2004; Quessy and Freedman, 2004) found that stimulation of the nucleus reticularis gigantocellularis (NRG) in monkey produced a consistent ipsilateral horizontal head rotation. In response, the eyes counter-rotated but with a gain of only −0.4, much lower than the nearly −1.0 that others report for passive head perturbations in primate (Dichgans et al., 1973; Newlands et al., 1999, 2001; Huterer and Cullen, 2002). The authors hypothesized that stimulation of NRG elicits a head movement command similar to that during eye-head gaze shifts, during which the gain of the VOR is thought to be attenuated. However, stimulation of paramedian pontine reticular formation (PPRF), which elicits a horizontal eye-head gaze shifts, produces gaze shifts of equal magnitude regardless of whether the head is restrained or unrestrained, suggesting that the VOR remains intact during these maneuvers (Gandhi et al., 2008). Likewise, stimulation of pontine omnipause neurons during eye-head gaze shifts, which prevents the eye saccade but allows the head to continue moving, reveals a near unity gain of ocular counter-rotation, adding further evidence that the VOR is not suppressed during eye-head gaze shifts but is fully functional (Gandhi and Sparks, 2007).

There are several limitations to our study. First, our lesioned animals appeared to have residual vestibular function (i.e., non-zero VOR gain). This may account for why the Cooperative Model still out-performed the Suppression Model despite our prediction that both would produce similar fits. Second, this was a relatively small study with only seven control and three lesioned animals, only two of which were we able to follow for more than 2 months after lesion. More animals followed for longer amounts of time would have allowed us to better characterize the process of recovery. Finally, our model was extremely simple, lacking dynamics as well as other known inputs to the vestibular system, including proprioception. The latter is of particular importance given the role the COR has been found to play in primates after labyrinthectomy. While we believe the COR most likely plays a similar role in the guinea pig, our goal in this paper was to simply address the contribution of PPEM to ocular stability. Further, studies would need to be done to assess the COR's role in gaze stabilization in this species.

Despite these limitations, our results suggest that not only are PPEM a part of normal gaze stabilization, but that they work cooperatively with the VOR. This is achieved by selectively cancelling the VOR during active head movements in a manner similar to Bizzi's linear summation theory. Further, it is precisely this selective cancellation that allows us to reconcile the conflicting reports in earlier literature as to whether PPEM are a part of normal gaze stabilization. Finally, it is interesting to note that while the gaze shifts Bizzi sought to explain are unique to foveate animals, we have found a similar phenomenon in an afoveate species during gaze stabilization, perhaps indicating that this system is a more primitive version of what is used in higher-order animals.

## Author contributions

SH: Performed experiments, analyzed data, prepared figures, drafted manuscript, edited and revised manuscript; WK: Edited and revised manuscript.

### Conflict of interest statement

The authors declare that the research was conducted in the absence of any commercial or financial relationships that could be construed as a potential conflict of interest.
